# Ookinete destruction within the mosquito midgut lumen explains *Anopheles albimanus* refractoriness to *Plasmodium falciparum* (3D7A) oocyst infection

**DOI:** 10.1016/j.ijpara.2011.12.005

**Published:** 2012-03

**Authors:** Luke A. Baton, Lisa C. Ranford-Cartwright

**Affiliations:** Wellcome Trust Centre for Molecular Parasitology, Institute of Infection, Immunity and Inflammation, College of Medical, Veterinary and Life Sciences, Sir Graeme Davies Building, 120 University Avenue, University of Glasgow, Glasgow G12 8TA, UK

**Keywords:** Bloodmeal, Malaria, Midgut, Mosquito, Ookinete, Oocyst, Parasite–vector interaction, Susceptibility

## Abstract

Previous studies have shown that the central American mosquito vector, *Anopheles albimanus*, is generally refractory to oocyst infection with allopatric isolates of the human malaria parasite *Plasmodium falciparum*. However, the reasons for the refractoriness of *A. albimanus* to infection with such isolates of *P. falciparum* are unknown. In the current study, we investigated the infectivity of the *P. falciparum* clone 3D7A to laboratory-reared *A. albimanus* and another natural vector of human malaria, *Anopheles stephensi*. *Plasmodium falciparum* gametocytes grown in vitro were simultaneously fed to both mosquito species and the progress of malaria infection compared. In 22 independent paired experimental feeds, no mature oocysts were observed on the midguts of *A. albimanus* 10 days after bloodfeeding. In contrast, high levels of oocyst infection were found on the midguts of simultaneously fed *A. stephensi*. Direct immunofluorescence microscopy and light microscopical examination of Giemsa-stained histological sections were used to identify when the *P. falciparum* clone 3D7A failed to establish mature oocyst infections in *A. albimanus*. Similar densities of macrogametes/zygotes, and immature retort-form and mature ookinetes were found within the bloodmeals of both mosquito species. However, in *A. albimanus*, ookinetes were seldom associated with the peritrophic matrix, and were neither observed in the ectoperitrophic space nor the midgut epithelium. In contrast, ookinetes were frequently observed in these midgut compartments in *A. stephensi*. Additionally, young oocysts were observed on the midguts of *A. stephensi* but not *A. albimanus* 2 days after bloodfeeding. Vital staining of the immature retort-form and mature ookinetes found within the luminal bloodmeal, demonstrated that a significantly greater proportion of these malaria parasite stages were non-viable in *A. albimanus* compared with *A. stephensi*. Overall, our observations indicate that ookinetes of the *P. falciparum* clone 3D7A are destroyed within the bloodmeal of *A. albimanus* and that the midgut lumen, rather than the midgut epithelium, is the site of mosquito refractoriness in this particular malaria parasite-mosquito vector combination.

## Graphical abstract

**Graphical abstract:**

**Figure:**
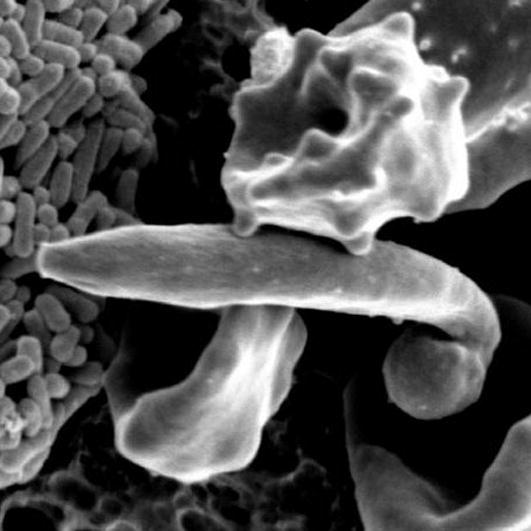

**Highlights:**

► Development of a human malaria parasite is compared between two mosquito vector species. ► Ookinete formation within the midgut lumen is equivalent in both mosquito species. ► *Plasmodium falciparum* fails to produce oocyst infections in *Anopheles albimanus*. ► All ookinetes are destroyed within the midgut lumen of *A. albimanus*. ► Malaria parasite survival in all of the different midgut compartments is quantified.

## 1 Introduction

*Anopheles albimanus* is an important vector of human malaria throughout central America (Pan American Health Organization, 1996). Accordingly, many previous studies have investigated the susceptibility of this mosquito species to infection with various malaria parasite species/strains. In general, these studies have shown that *A. albimanus* is markedly more refractory to mature oocyst infection than other mosquito species that have been investigated: many malaria parasite species/strains fail to establish mature oocyst infections in *A. albimanus* while malaria parasites to which this mosquito species is susceptible often exhibit comparatively low levels of mature oocyst infection (e.g. Hunninen, 1953; Eyles, 1960; Omar, 1968a; Collins et al., 1969, 1999; Bafort, 1971; Collins and Contacos, 1980; Nayar and Young, 1984; Vaughan et al., 1991, 1994a; Grieco et al., 2005; Frischknecht et al., 2006). With regard to the human malaria parasite species *Plasmodium falciparum* and *Plasmodium vivax*, *A. albimanus* tends to be susceptible only to infection with naturally-encountered (i.e. coindigenous or sympatric) malaria parasite species/strains, implying a relatively high degree of specificity in the relationship between malaria parasites and this mosquito species (Boyd et al., 1938; Boyd and Jobbins, 1940; Jeffery et al., 1950, 1954; Eyles, 1960; Warren and Collins, 1981; Teklehaimanot et al., 1987; Vaughan et al., 1994b; Gonzalez-Ceron et al., 1999, 2007; Rodriguez et al., 2000; Li et al., 2001; Joy et al., 2008). Furthermore, there are considerable differences in susceptibility to malaria infection between different strains of *A. albimanus*, indicating the existence of complex interactions determining parasite–vector compatibility (Warren et al., 1977, 1979; Chan et al., 1994; Gonzalez-Ceron et al., 2000; Grieco et al., 2005). In contrast, other mosquito species including *Anopheles stephensi*, an important vector of human malaria in central and southern Asia, exhibit broad and moderately high susceptibility to infection with various malaria parasite species/strains regardless of their geographical origin (e.g. Omar, 1968a; Collins et al., 1969, 1999; Collins and Contacos, 1980; Warren and Collins, 1981; Vaughan et al., 1994a; Grieco et al., 2005; Frischknecht et al., 2006). Although there are exceptions to this broad pattern, these observations imply that in general some mosquito species are inherently more refractory, and others inherently more susceptible, to malaria parasite infection than other mosquito species (Warren and Collins, 1981).

The reasons for the general refractoriness of *A. albimanus* to oocyst infection with malaria parasites are largely unknown, and have seldom been investigated in detail, with two notable exceptions (Omar, 1968b; Gonzalez-Ceron et al., 2001). Malaria parasites undergo a complex series of developmental and migratory transitions within the mosquito vector, starting with ingested gametocytes transforming into gametes that fertilise to produce zygotes within the bloodmeal, which subsequently transform into motile ookinete stages that migrate from the midgut lumen, across the midgut epithelium, and eventually develop into sessile oocyst stages on the basal (outer) surface of the midgut epithelium (reviewed in Baton and Ranford-Cartwright, 2005). The failure to successfully complete any one (or more) of these developmental and/or migratory transitions presumably accounts for the relatively low levels of mature oocyst infection observed in *A. albimanus*. Previous studies using a variety of human and non-human malaria parasite species have observed normal development of mature ookinetes within the midgut lumen of *A. albimanus* and have consequently concluded that the success of the ookinete-to-oocyst transition is the key determinant of the susceptibility of *A. albimanus* to malaria parasite infection (Eyles and Young, 1950; Vaughan et al., 1991, 1994a,b; Chege et al., 1996). Although the precise time at which ookinetes failed to establish infection in *A. albimanus* was not identified in these previous studies, the midgut epithelium was proposed to be the site of mosquito resistance to malaria parasite infection (Vaughan et al., 1994a). However the absence, or low levels, of mature oocyst infection observed in *A. albimanus* could have resulted from ookinete destruction prior to or after, rather than during, invasion of the midgut epithelium. Indeed, there is evidence that the refractoriness of *A. albimanus* to malaria parasite infection may result from multiple mechanisms operating in different compartments of the mosquito midgut. For example, Gonzalez-Ceron et al. (2001) reported loss of *P. vivax* ookinetes both before and during ookinete invasion of the midgut epithelium with subsequent developmental arrest of those malaria parasites that had survived to become oocysts, while Omar (1968b) reported destruction of the ookinetes of the non-human primate malaria parasite *Plasmodium cynomolgi bastianelli* within the midgut lumen during the process of bloodmeal digestion.

Previous studies using the *P. falciparum* isolate NF54 (and various unspecified clones derived therefrom) observed very low levels of mature oocyst infection in *A. albimanus* compared with other susceptible mosquito species (Vaughan et al., 1994b; Chege et al., 1996; Grieco et al., 2005). However, the reasons for the low level of mature oocyst infection in *A. albimanus* with human malaria parasites derived from this isolate remain unknown (Chege et al., 1996; Chege and Beier, 1998). In the current study, we compared the susceptibility of *A. albimanus* and *A. stephensi* to infection with the *P. falciparum* clone 3D7A, which was previously derived from the NF54 isolate by limiting dilution (Walliker et al., 1987). We found that the *P. falciparum* clone 3D7A, which is highly infectious to *A. stephensi*, was unable to establish mature oocyst infections in *A. albimanus*. Using various microscopical techniques, we identified the endoperitrophic space of the midgut lumen, rather than the midgut epithelium, as the site of refractoriness of *A. albimanus* to the 3D7A clone of *P. falciparum*. Our observations and those of others (Omar, 1968b; Gonzalez-Ceron et al., 2007) call into question the general and unexamined assumption that the midgut epithelium is the major barrier to ookinete infection of the mosquito vector.

## 2 Materials and methods

### 2.1 Parasites and mosquitoes

The *P. falciparum* clone 3D7A was cultured in vitro under conditions permissive for the development of mature gametocytes infective to mosquitoes, as described in detail elsewhere (Ifediba and Vanderberg, 1981; Ponnudurai et al., 1982; Carter et al., 1993). The NF54 isolate from which the 3D7A clone is derived (Walliker et al., 1987) was isolated from a 10 year-old girl in 1979 who lived near Schiphol airport, Amsterdam, the Netherlands, and who had never been outside the Netherlands (Delemarre and van der Kaay, 1979; Ponnudurai et al., 1981). As autochthonous transmission of human malaria was not known to have occurred, this case was assumed to have arisen by the introduction, from an incoming flight to Schiphol airport, of a *P. falciparum*-infected mosquito from a malaria endemic region (Delemarre and van der Kaay, 1979). Therefore, the ultimate geographical origin of the *P. falciparum* isolate NF54 is unknown.

Stock colonies of *A. albimanus* and *A. stephensi* were maintained as previously described (Baton and Ranford-Cartwright, 2004). The *A. albimanus* Panama strain used originated from a colony established at the London School of Hygiene and Tropical Medicine (LSHTM), United Kingdom, in 1986, which was itself founded from a colony held at the Centre for Disease Control, Atlanta, Georgia USA (Patricia Aiyenuro, LSHTM, personal communication). The *A. stephensi* SDA-500 strain was obtained from Imperial College, London, UK, and originates from the Sind strain selected for susceptibility to *P. falciparum* infection, with some outcrossing to the Kasur strain (Feldmann and Ponnudurai, 1989).

### 2.2 *Plasmodium falciparum* infection of *A. albimanus* and *A. stephensi*

Five to 7 days after emergence from pupae, adult females of both mosquito species were simultaneously membrane-fed with uninfected human blood mixed with cultured gametocyte stages of *P. falciparum* according to standard procedures (Ponnudurai et al., 1982; Carter et al., 1993).

### 2.3 Direct immunofluorescence microscopy

The FITC-conjugated anti-Pfs25 monoclonal antibody (mAb) 32F71 (Vermeulen et al., 1985) was used to monitor and compare the development of the *P. falciparum* clone 3D7A within *A. albimanus* and *A. stephensi* according to previously published protocols (Robert et al., 1995; Gouagna et al., 1998). Pfs25 is specifically expressed on the surface of macrogametes and zygotes approximately 2–4 h after their formation and throughout the subsequent stages of malaria parasite development until the mid/late oocyst stage (Vermeulen et al., 1985; Lensen et al., 1992).

A modified version of the technique of Chege and Beier (1994) was used to monitor the development of “round forms” (unfertilised macrogametes and zygotes, which are indistinguishable using this technique), and immature retort-form and mature ookinetes within the bloodmeal. Dissected midguts were individually homogenised in 320 μl of PBS and 20 μl of the resulting midgut homogenate spotted onto Teflon®-printed microwell glass slides (VWR International, UK) previously treated with 3-aminopropyltriethoxysilane (APES) according to the manufacturer’s instructions (Sigma, UK). Slides were allowed to air-dry, fixed in ice cold acetone for 2 min, air-dried again and placed into a desiccator for at least 24 h, before storing at −25 °C.

To assay ookinete invasion of the midgut epithelium, paraformaldehyde-fixed midgut epithelial sheets were prepared for immunofluorescence as previously described for rodent malaria parasites in anopheline mosquitoes (Han et al., 2000).

For immunofluorescent detection of early oocysts on the basal surface of the midgut epithelium, midguts were dissected between 56 and 62 h post-bloodfeeding (pbf), and whole mounts of unfixed midguts prepared as previously described (Gouagna et al., 1999).

Vital staining of round forms, and immature retort-form and mature ookinetes was performed by homogenising freshly dissected unfixed midguts and then incubating for 30 min at room temperature in 20 μl of FITC-labelled mAb 32F71 in PBS containing 2.0 mg/ml of propidium iodide (PI) (Sigma) and 0.5 mg/ml Hoechst 33258 (HO) (Sigma). PI is a cell-impermeant fluorescent dye that stains both RNA and DNA, and is generally excluded from viable cells but can enter dying/dead cells possessing a compromised plasma membrane. In contrast, HO is a cell-permeant, DNA-specific fluorescent dye that can penetrate live, viable cells possessing an intact plasma membrane. The different absorption and emission spectra of PI and HO enable use of the two dyes in triple staining experiments, together with the FITC-labelled mAb 32F71, to assess viability of Pfs25-positive parasite stages: live cells stain only with HO whilst dying/dead cells stain with both HO and PI.

For the above assays, all samples were mounted under a cover slip and examined under epifluorescence using a Zeiss Axioskop light microscope fitted with a 100 W mercury-arc lamp and Zeiss filter sets 02, 09 and 15.

### 2.4 Determination of total midgut protein content during bloodmeal digestion

The total protein content of the homogenised midgut samples used to monitor malaria parasite development within the bloodmeal (see Section 2.3) was estimated using a bicinchoninic acid (BCA) protein assay kit, according to the manufacturer’s instructions (Sigma). The protein content of the midgut samples was estimated by comparison with a standard curve of known concentrations of BSA diluted in PBS. Fifty microlitres of each sample were added in duplicate to 96-well flat-bottomed assay plates (Sigma). Two hundred microlitres of BCA working reagent were then added to each well, the assay plate sealed and incubated at 37 °C for 30 min. Plates were subsequently read at a wavelength of 570 nm using an MRX Revelation Plate Reader (Dynex Technologies, Inc., USA).

### 2.5 Preparation and examination of histological sections

Preparation of the 2 μm-thick, Giemsa-stained histological sections from blood-fed mosquito midguts were prepared and examined as previously described (Baton and Ranford-Cartwright, 2004). Each midgut was completely sectioned and all of the resulting histological sections for each midgut were examined using bright-field light microscopy at 400 and 1000× magnification.

### 2.6 Statistical analysis

All statistical analyses were performed using XLSTAT version 7.5.2 (Addinsoft, Inc., 1995–2004).

## 3 Results

### 3.1 The prevalence and intensity of *P. falciparum* oocyst infection at day 10 pbf

Cultured gametocytes of *P. falciparum* clone 3D7Awere simultaneously fed to *A. albimanus* and *A. stephensi* in 22 independent paired experimental feeds (Table 1). Overall, a total of 553 *A. albimanus* were dissected and examined for mature oocysts at day 10 pbf. No oocysts were observed on any of the *A. albimanus* midguts examined. In contrast, 512 of the 645 (79.1%) *A. stephensi* midguts examined at day 10 pbf were infected with at least one oocyst, demonstrating the infectivity of the gametocytes fed to *A. albimanus*. Overall, 14,404 oocysts were observed in the *A. stephensi* examined, with a median intensity of 18.5 oocysts per infected midgut. The median prevalence and the median intensity of oocyst infection at day 10 pbf, per experimental feed, were both highly significantly different between *A. albimanus* and *A. stephensi* (two-tailed Mann–Whitney *U* test, *z*_0.05, 1.960_ = 6.074, *P* < 0.0001 and *z*_0.05, 1.960_ = 6.072, *P* < 0.0001, respectively) (Table 1).

### 3.2 Direct immunofluorescence microscopy of malaria parasite development

**Fig. 1:**
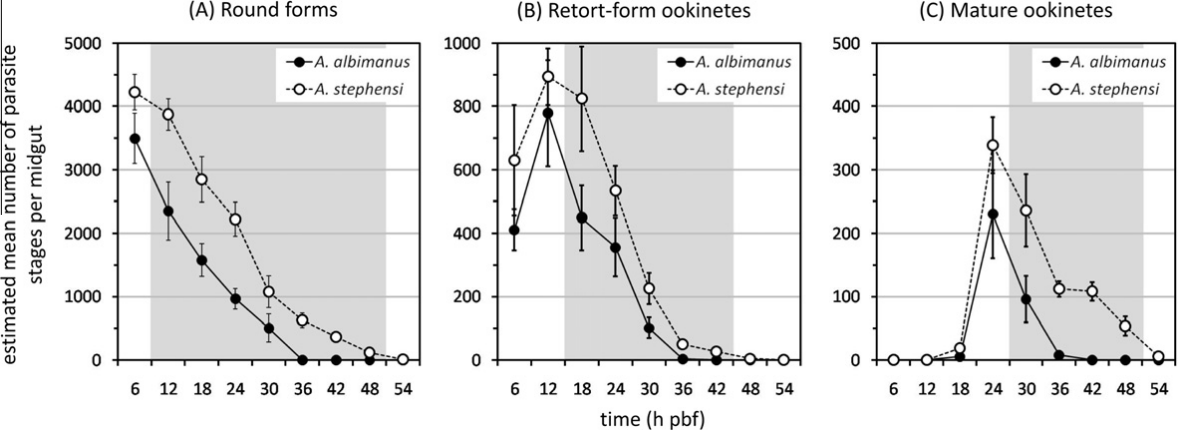
Comparison of the densities and kinetics of the three developmental stages (A–C) of *Plasmodium falciparum* clone 3D7A occurring within the midgut lumens of *Anopheles albimanus* and *Anopheles stephensi*. Values shown are arithmetic means and their associated standard errors. For each mosquito species, midguts from six individuals were assayed for each time point (h post-bloodfeeding (pbf)). The shaded area indicates time points that differed significantly between the two mosquito species (*t* test, *P* < 0.05). The data shown are for one experiment. A second experiment gave similar results, showing the same general pattern of change over time in malaria parasite densities, although the absolute numbers of malaria parasites were lower (data not shown).

**Fig. 2:**
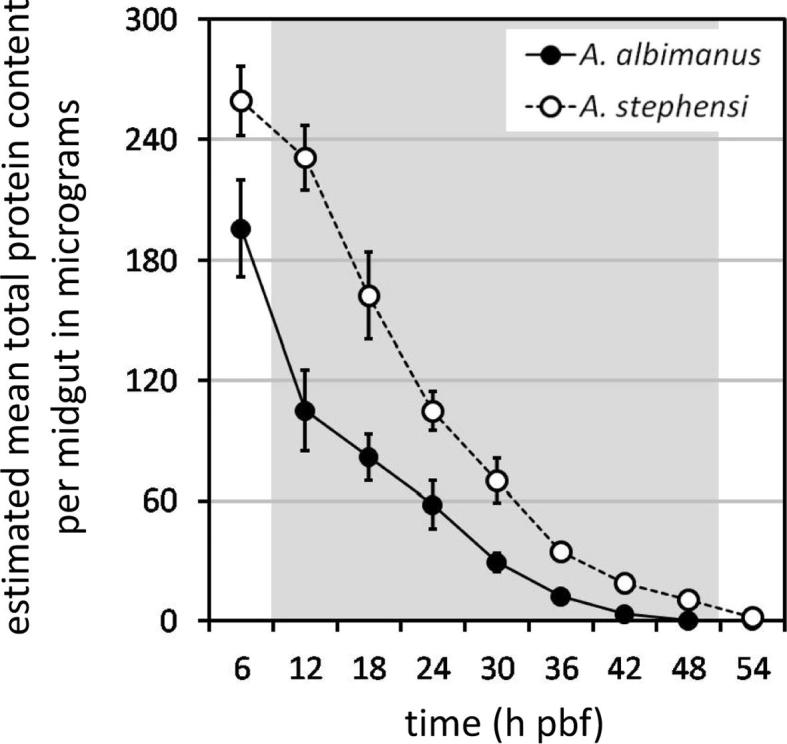
Comparison of the rate of bloodmeal digestion between *Anopheles albimanus* and *Anopheles stephensi*. Values shown are arithmetic means and their associated standard errors. The midguts assayed were the same as those presented in Fig. 1, so that this figure is directly comparable with the graphs in Fig. 1. The shaded area indicates time points (h post-bloodfeeding (pbf)) that differed significantly between the two mosquito species (*t* test, *P* < 0.05). The second experiment described in the Fig. 1 legend gave comparable results (data not shown).

Development of *P. falciparum* clone 3D7A within the bloodmeals of *A. albimanus* and *A. stephensi* was compared in two independent experimental feeds (Fig. 1). The formation of round forms, immature retort-form ookinetes and mature ookinetes within the bloodmeal was similar in both mosquito species, with regard to both the timing and the number of malaria parasite stages formed (Fig. 1). The density of round forms was highest immediately after bloodfeeding and declined consistently thereafter with increasing time pbf (Fig. 1A), paralleling the decline in the mean total protein content per midgut (Fig. 2). The density of retort-form ookinetes was initially low, rose to a peak at 12 h pbf and then declined (Fig. 1B), while mature ookinetes were first observed at 18 h pbf, with densities peaking at 24 h pbf before declining thereafter (Fig. 1C). The peak densities of round forms (6 h pbf), retort-form ookinetes (12 h pbf) and mature ookinetes (24 h pbf) did not differ significantly between *A. albimanus* and *A. stephensi* (*t* test, *P* > 0.05 in all instances). However, with increasing time pbf, the density of the three distinct developmental stages of the malaria parasite differed significantly between the two mosquito species, being markedly lower in *A. albimanus* compared with *A. stephensi*. In particular, the densities of retort-form and mature ookinetes were both significantly lower within the bloodmeals of *A. albimanus* from, respectively, 18 and 30 h pbf onwards (*t* test, *P* < 0.05 in all instances) (Fig. 1B and C). Comparison of the mean total protein content per midgut with increasing time pbf indicated that bloodmeal digestion occurred more rapidly and was completed sooner in *A. albimanus* than *A. stephensi* (Fig. 2).

In three paired experimental feeds, attempts to identify ookinetes between 24 and 32 h pbf in paraformaldehyde-fixed midgut epithelial sheets (*n* = 48 for both mosquito species), using the anti-Pfs25 mAb 32F71, were unsuccessful, despite heavy oocyst infections at day 10 pbf in the same cohorts of *A. stephensi*. The anti-Pfs25 mAb 32F71 used is apparently unable to recognise paraformaldehyde-fixed ookinetes (data not shown), consistent with previous reports that the epitope recognised by this mAb is conformation-dependent (Will Roeffen, Department of Medical Microbiology, Nijmegen Center for Molecular Life Science, the Netherlands, personal communication; Fries et al., 1989).

**Fig. 3:**
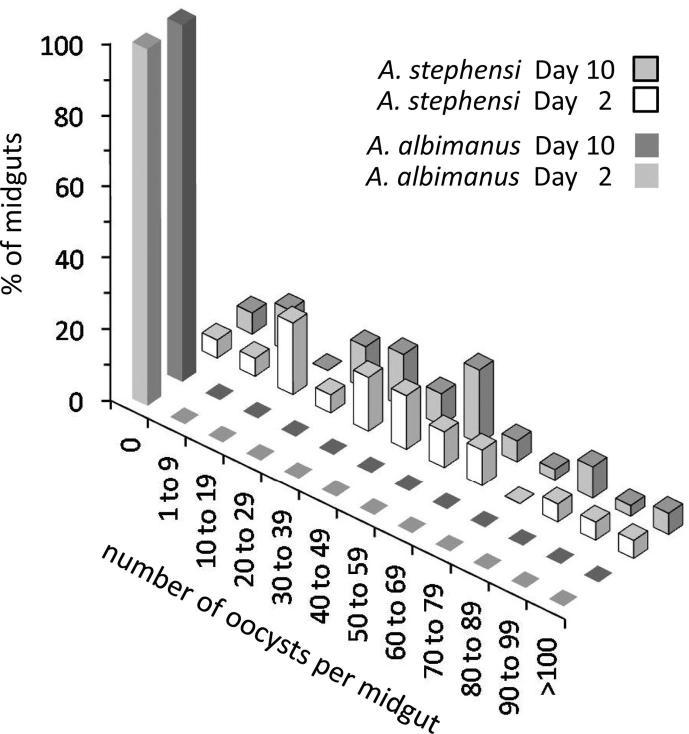
Comparison of the distribution of early and late oocysts of *Plasmodium falciparum* clone 3D7A in *Anopheles albimanus* and *Anopheles stephensi*. The bar chart illustrates the distribution of early (day 2 post-bloodfeeding (pbf)) and late (day 10 pbf) oocysts. No oocysts were observed in *A. albimanus*, while the distribution of oocysts in *A. stephensi* was not significantly different between days 2 and 10 pbf. Data shown are those from Experiment 2 summarised in Table 2.

The prevalence and intensity of early oocyst infection in *A. albimanus* and *A. stephensi* were compared in three independent paired experimental feeds (Table 2, Fig. 3). Overall, a total of 71 *A. albimanus* were dissected and examined at day 2 pbf for early oocysts. However, no oocysts were observed on any of the *A. albimanus* midguts examined. In contrast, 56 of the 62 (90.3%) *A. stephensi* midguts examined at day 2 pbf were infected with at least one oocyst. The prevalence and median intensity of oocyst infection at day 2 pbf, for each experimental feed, differed significantly between *A. albimanus* and *A. stephensi* (Table 2). There was no significant difference in either the prevalence or intensity of oocyst infection at days 2 and 10 pbf within the same cohorts of infected *A. stephensi* (two-tailed Fisher’s Exact test, *P* > 0.660 and two-tailed Mann–Whitney *U* test, *z*_0.05, 1.960_ > −1.304, *P* > 0.598, respectively) implying that all ookinetes which transformed into oocysts survived to complete sporogony (Table 2, Fig. 3).

### 3.3 Giemsa-stained histological sections of *P. falciparum*-infected-blood-fed midguts

**Fig. 4:**
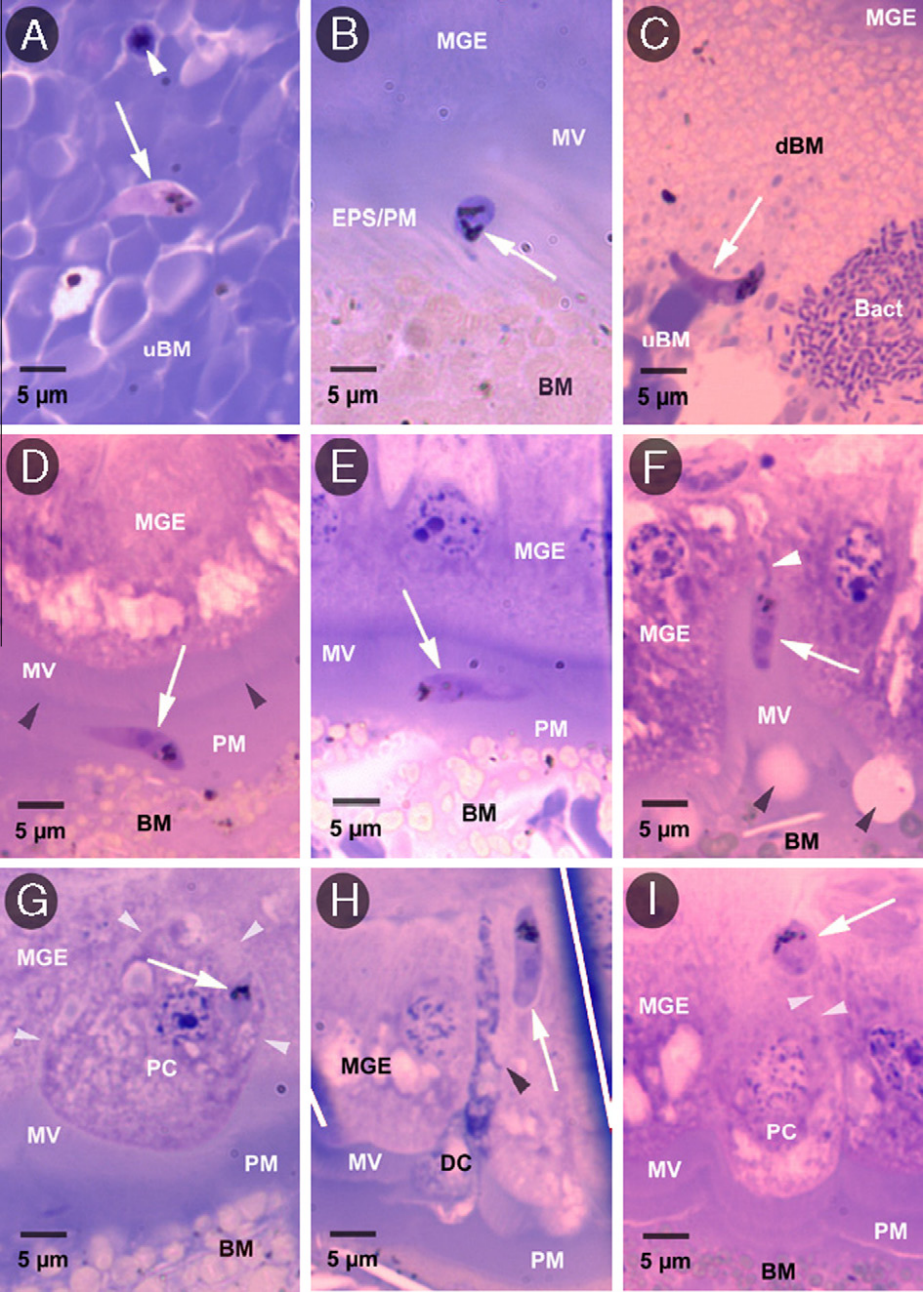
Histological sections illustrating the locations of *Plasmodium falciparum* clone 3D7A parasites observed within the midguts of *Anopheles albimanus* and *Anopheles stephensi*. (A and B) Ookinetes located in different regions of the midgut of *A. albimanus*. (A) Ookinete (white arrow) within the inner, central undigested region of the bloodmeal (uBM), surrounded by intact uninfected erythrocytes. An intra-erythrocytic asexual (schizont) stage parasite is also visible (white arrowhead). (B) One of three ookinetes observed in *A. albimanus* associated with the endoperitrophic side of the peritrophic matrix (PM). BM, bloodmeal; EPS, ectoperitrophic space; MV, microvillar brush border of the midgut epithelium (MGE). (C–I) Malaria parasites located in different regions of the midgut of *A. stephensi*. (C) Ookinete (white arrow) in the transitional zone between the inner, central uBM and the peripheral digested region of the bloodmeal (dBM). A small cluster of bacilli (bact) is located close to the ookinete. (D) Ookinete (white arrow) within the PM. Dark arrowheads indicate EPS. (E) Ookinete (white arrow) within the ectoperitrophic space, situated between the PM and the MV of the ME. (F) Ookinete entering the ME. The majority of the ookinete is extracellular (white arrow) within the MV of the MGE, while the anterior portion of the ookinete, which presents the narrow, constricted “stalk-form” morphology, resides within the MGE (white arrowhead). Dark arrowheads indicate artifactual “blebbing” of the apical plasma membranes of the midgut epithelial cells. (G) An intracellular ookinete (white arrow) located within a midgut epithelial cell (PC) that protrudes into the midgut lumen, but which is otherwise morphologically normal. White arrowheads delineate the PC. (H) An apparently intercellular ookinete (white arrow) located between morphologically normal midgut epithelial cells, but in close proximity to a flaccid and abnormally dark staining midgut epithelial cell (DC), through which the ookinete is presumed to have migrated intracellularly prior to attaining its intercellular location. The ookinete is apparently situated where three adjacent midgut epithelial cells converge (dark arrowhead). White lines indicate artifactual creases in the histological section. (I) Oocyst (white arrow) on the basal surface of the MGE, located immediately above the lateral plasma membranes of the underlying midgut epithelial cells (white arrowheads). One of the underlying PC appears morphologically normal, but protrudes slightly from the plane of the MGE into the midgut lumen. All images are 1000× magnification.

In order to compare ookinete migration from the bloodmeal and invasion of the midgut epithelium in the two mosquito species, Giemsa-stained histological sections were prepared from the midguts of *A. albimanus* and *A. stephensi* collected between 24 and 48 h pbf in two independent paired experimental feeds (Table 3). As previously reported, ookinetes were frequently observed during all stages of migration to, and across, the midgut epithelium of *A. stephensi* (Fig. 4C–H, Table 3) (Baton and Ranford-Cartwright, 2004). In contrast, in *A. albimanus*, ookinetes were only very rarely observed on the internal bloodmeal (endoperitrophic) side of the peritrophic matrix (Fig. 4B), and were not observed in either the ectoperitrophic space or within the midgut epithelium (Table 3). Furthermore, young oocysts were frequently observed in *A. stephensi* but were never seen in *A. albimanus* (Fig. 4I, Table 3). When the number of malaria parasite stages observed in each location of the midgut histological sections were compared between the two mosquito species, the difference was highly significant (two-tailed Mann–Whitney *U* test, *z*_0.05, 1.960_ < −2.795, *P* < 0.005 for all three locations) (Table 3). Consistent with the absence of malaria parasite infection of the midgut epithelium of *A. albimanus*, the pathology of the midgut epithelium associated with ookinete invasion in *A. stephensi* (Fig. 4G–I) (Baton and Ranford-Cartwright, 2004) was not observed in *A. albimanus*.

Examination of the histological sections did reveal two notable differences between the midguts of *A. albimanus* and *A. stephensi*. First, the progression of bloodmeal digestion differed markedly between the two mosquito species. In mosquitoes, bloodmeal digestion proceeds inwards from the midgut epithelium towards the midgut lumen and from the posterior to the anterior of the bloodmeal (our observations and Huff, 1934; Graf et al., 1986). At equivalent times pbf, the peripheral zone of digested blood was considerably greater in *A. albimanus* compared with *A. stephensi* and contained an appreciably smaller inner mass of undigested erythrocytes. This difference in bloodmeal digestion was apparent at 24 h pbf, when the digested region of the bloodmeal in *A. stephensi* was still predominantly at the periphery and the inner central mass remained undigested, while the posterior half of the bloodmeal in *A. albimanus* had been digested. These histological differences in bloodmeal digestion between the two mosquito species are consistent with the differences in the decline of the total midgut protein content with increasing time pbf in *A. albimanus* and *A. stephensi* (Fig. 2). The second notable difference between the two mosquito species was the abundance of bacteria within the midgut lumen. Large bacterial aggregates were observed in all of the *A. albimanus* midguts examined. These bacterial aggregates were located in the anterior-most, undigested region of the bloodmeal and were usually located within the peritrophic matrix surrounding the bloodmeal, confined entirely within the endoperitrophic space. Bacteria were generally absent or only sparsely and individually scattered throughout the peripheral and posterior digested regions of the bloodmeal. In marked contrast, most of the *A. stephensi* midguts examined were free of bacteria, although small clusters were occasionally found in a few midguts (Fig. 4C). From the histological sections and from scanning electron microscopy (unpublished observations), the majority of bacteria present within the midgut lumen of *A. albimanus* were found to be bacilli, cocci being only very rarely observed.

### 3.4 Estimation of ookinete mortality rates within different midgut compartments

**Fig. 5:**
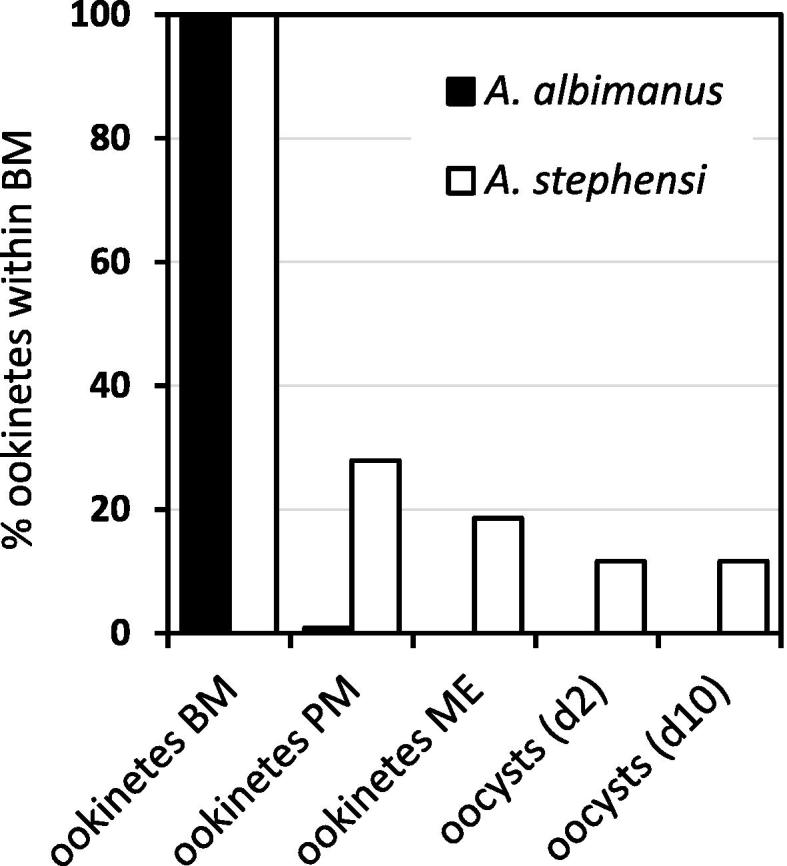
Estimates of *Plasmodium falciparum* clone 3D7A survival in different midgut compartments of *Anopheles albimanus* and *Anopheles stephensi* during the ookinete-to-oocyst transition. The bar chart illustrates the percentage of ookinetes per mosquito that develop within the bloodmeal (BM), which subsequently migrate to different midgut compartments and transform into oocysts. These estimates of ookinete/oocyst survival were derived from the data presented in Figs. 1 and 3, and Tables 1–3. Ookinetes BM, ookinetes within the bloodmeal; ookinetes PM, ookinetes associated with the peritrophic matrix; ookinetes ME, ookinetes associated with the midgut epithelium (not including extracellular ookinetes within the microvillar brush border); oocysts (d2), oocysts 2 days post-bloodfeeding; and oocysts (d10), oocysts 10 days post-bloodfeeding.

Combining the data from the different experimental feeds reported above, ookinete and oocyst mortality rates within different compartments of the midgut were calculated for the *P. falciparum* clone 3D7A in *A. albimanus* and *A. stephensi* (Fig. 5). In *A. albimanus*, less than 1% of ookinetes forming within the bloodmeal attained the peritrophic matrix, while none survived to enter the ectoperitrophic space, indicating that all ookinetes forming within the bloodmeal were lost within the endoperitrophic space (Fig. 5). In *A. stephensi*, approximately 72.1% of all ookinetes forming within the bloodmeal were lost within the endoperitrophic space and failed to attain the peritrophic matrix; 8.6% were lost between penetration of the peritrophic matrix and entry into the midgut epithelium; and 8.0% were lost during invasion of the midgut epithelium. The remaining 11.2% of all ookinetes forming within the bloodmeal successfully transformed into oocysts on the basal surface of the midgut epithelium (Fig. 5). The inferred mortality of ookinetes during invasion of the midgut epithelium (approximately 40%) is quantitatively proportional to the previously reported number of morphologically-abnormal midgut epithelial cells not associated with detectable malaria parasites, consistent with the loss of these malaria parasites through lysis during or after invasion of midgut epithelial cells (Baton and Ranford-Cartwright, 2004).

### 3.5 Malaria parasite viability within the bloodmeal

**Fig. 6:**
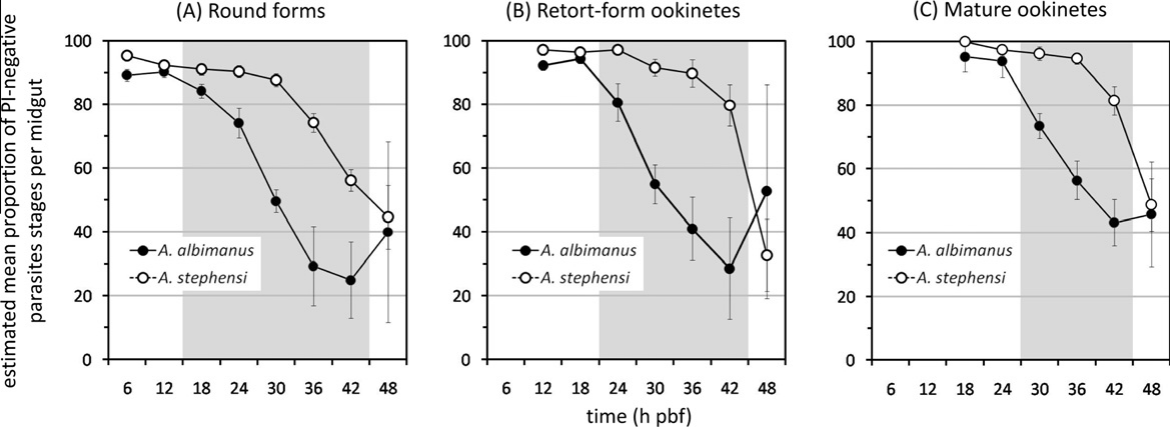
Comparison of the viability of the three developmental stages (A–C) of *Plasmodium falciparum* clone 3D7A occurring within the midgut lumens of *Anopheles albimanus* and *Anopheles stephensi*. Values shown are arithmetic means of the proportions of propidium iodide (PI)-negative (viable) malaria parasites and their associated 95% confidence intervals. For each mosquito species, midguts from 12 individuals were assayed for each time point (h post-bloodfeeding). For each developmental stage, 50–100 parasites were counted per midgut or, for later time points when parasite numbers declined, as many parasites as were present. The data are pooled from three independent experimental feeds. The shaded area indicates time points that differed significantly between the two mosquito species (*t* test, *P* < 0.05).

In order to further characterise the causes of *P. falciparum* clone 3D7A loss within *A. albimanus* and *A. stephensi*, the viability of the three different malaria parasite developmental stages present within the midgut lumen were compared using vital staining in three independent experimental feeds (Fig. 6). Within approximately the first 24 h pbf, the majority of round forms, and immature retort-form and mature ookinetes were negative for PI in both mosquito species, implying that most of these malaria parasite stages were viable during this period. Furthermore, the proportion of PI-positive parasites did not differ significantly between the two mosquito species during this time (Fig. 6). However from approximately 24 h pbf onwards, the proportion of malaria parasites positive for PI was significantly higher in *A. albimanus* than *A. stephensi* for each of the three different developmental stages (Fig. 6), indicating reduced viability of these malaria parasite stages in the midgut lumen of *A. albimanus* during this period.

## 4 Discussion

The central American mosquito vector *A. albimanus* is generally refractory to oocyst infection with allopatric isolates of the human malaria parasite *P. falciparum* (Boyd et al., 1938; Boyd and Jobbins, 1940; Eyles and Young, 1950; Jeffery et al., 1950), but the mechanism of refractoriness to this malaria parasite species is unknown. In the current study, we investigated the infectivity of *P. falciparum* clone 3D7A to laboratory-reared *A. albimanus* Panama strain and found that ookinetes failed to migrate from the bloodmeal and invade the midgut epithelium, and were apparently destroyed within the endoperitrophic space before the peritrophic matrix was attained, during the period of ookinete invasion of the midgut epithelium in simultaneously-fed and susceptible *A. stephensi*. Almost all (>99%) of the ookinetes forming within the bloodmeal of *A. albimanus* failed to reach the peritrophic matrix, while significant numbers of ookinetes (>72%) were also lost in *A. stephensi* despite the relatively high susceptibility of the latter mosquito species to malaria oocyst infection. In contrast, both the peritrophic matrix (Omar, 1968b; Meis and Ponnudurai, 1987; Ponnudurai et al., 1988) and the midgut epithelium (Vaughan et al., 1994a; Shahabuddin et al., 1995; Kaplan et al., 2001) have previously been suggested to be the major sites of attrition in malaria parasite densities during infection of the mosquito. We observed no accumulation of ookinetes at the peritrophic matrix in either *A. albimanus* or *A. stephensi*, indicating that it is not a barrier to *P. falciparum* clone 3D7A infection. However, attempts to confirm this conclusion by disrupting the peritrophic matrix via addition of exogenous chitinase to the infectious gametocyte-containing bloodmeal as previously reported (Shahabuddin et al., 1993, 1995; Villalon et al., 2003) were unsuccessful: the peritrophic matrix seemingly remained intact, and there was no effect on the level of *P. falciparum* 3D7A oocyst infection in either mosquito species (data not shown). Overall, our observations suggest that the midgut lumen, rather than either the peritrophic matrix or the midgut epithelium, is the major barrier to *P. falciparum* clone 3D7A infection of *A. albimanus*. A number of different factors might reduce ookinete densities within the midgut lumen of both *A. albimanus* and *A. stephensi*, including midgut digestive proteases, any microbial flora within the midgut lumen and mosquito immune responses.

Digestive proteases secreted from the midgut epithelium following bloodmeal ingestion can cause ookinete destruction (Gass, 1977, 1979; Gass and Yeates, 1979; Yeates and Steiger, 1981). In our studies, bloodmeal digestion was completed more rapidly in *A. albimanus* than *A. stephensi*, and the parallel decline in round form densities and total protein content per midgut suggest that at least the loss of these malaria parasite stages is related to differences in the rate of bloodmeal digestion between the two mosquito species. Omar (1968b) made similar observations, arguing that differences in the intensity of bloodmeal digestion explained the differential susceptibility of *A. albimanus* and *A. stephensi* to *P. cynomolgi bastianellii*. However, absolute levels of digestive protease activity in *A. albimanus*, *Anopheles freeborni* and *Anopheles gambiae* did not correlate with differences in susceptibility to infection with *P. falciparum* isolate NF54 (Chege et al., 1996; Chege and Beier, 1998), while the absolute peak activity levels of the digestive enzymes, trypsin and chymotrypsin, were found to be similar in *A. albimanus* and *A. stephensi* (Hörler and Briegel, 1995). The peak digestive protease levels were, however, attained earlier in *A. albimanus* than *A. stephensi* (14 versus 30, and 20 versus 36, h pbf, respectively, for trypsin and chymotrypsin) (Hörler and Briegel, 1995), and it is possible that temporal differences in peak digestive protease activity between *A. albimanus* and *A. stephensi* are responsible for the increased loss of ookinetes in *A. albimanus*.

The microbial flora present within the mosquito midgut lumen can inhibit the development of oocyst infection in mosquitoes (Micks and Ferguson, 1961; Pumpuni et al., 1993, 1996; Beier et al., 1994; Luckhart et al., 1998; Gonzalez-Ceron et al., 2003; Frischknecht et al., 2006). The marked difference we observed in the number of bacteria in the midgut lumens of *A. albimanus* and *A. stephensi* might, therefore, account for the differences in *P. falciparum* clone 3D7A oocyst infection between these two mosquito species. However, addition of the antibiotic gentamicin to the mosquito’s sugar solution prior to bloodfeeding as previously reported (Beier et al., 1994; Luckhart et al., 1998; Frischknecht et al., 2006), had no effect on the level of oocyst infection in either mosquito species (data not shown). Furthermore, we have found that other clones of *P. falciparum* derived from central and South American isolates are capable of establishing oocyst infections in our *A. albimanus* colony (data not shown). Taken together, these observations suggest that the bacteria present in the midgut lumen of *A. albimanus* are not responsible for the refractoriness of this mosquito species to infection with the *P. falciparum* clone 3D7A.

In laboratory systems, mosquito immune responses are an important determinant of *Plasmodium* infection after ookinete invasion of the midgut epithelium (reviewed in Christophides et al., 2004; Baton et al., 2008). However, whether such mosquito immune responses are also active in the midgut lumen and can act as anti-parasitic effectors within the endoperitrophic space is not currently known. An increasing number of studies indicate that microbial flora in the mosquito midgut lumen can trigger anti-bacterial immune responses capable of limiting malaria parasite infection (Luckhart et al., 1998; Molina-Cruz et al., 2008; Meister et al., 2009; Kumar et al., 2010), but the effect (if any) of such immune responses on ookinetes within the midgut lumen has not been determined. A trypanolytic midgut glycoprotein has been isolated from *A. albimanus*, but its effect on malaria parasites has not been investigated (Nok et al., 2002).

Numerous previous studies have investigated the population dynamics of malaria parasite development within the mosquito by comparing the mortality between different parasite life cycle stages (reviewed in Vaughan, 2007). We believe that our study provides the first estimates of the proportion of ookinetes forming within the bloodmeal that attain the peritrophic matrix, subsequently reach the ectoperitrophic space and the microvillar brush border of the midgut epithelium, and then succeed in completing invasion across the midgut epithelium, enabling quantitative estimates of ookinete mortality in all of the different compartments of the mosquito midgut. Distinct losses of ookinetes occur within both the endo- and ectoperitrophic spaces of the midgut lumen, as well as within the midgut epithelium. Significantly, survival of *P. falciparum* clone 3D7A ookinetes during invasion of the *A. stephensi* midgut epithelium was relatively high (approximately 60%). Our observations and those of others reporting loss of ookinetes within the endoperitrophic space (Huff, 1934; Omar, 1968b; Sluiters et al., 1986; Al-Olayan et al., 2002; Gonzalez-Ceron et al., 2007) have potentially significant implications for transmission-blocking control strategies which currently consider ookinete invasion of the midgut epithelium as the “weakest link” in malaria parasite development within the mosquito vector because parasite densities are lowest during this period (e.g. Christophides, 2005). The greater mortality of ookinetes within the midgut lumen observed here, especially within the endoperitrophic space, together with the relatively high efficiency of ookinete invasion of the midgut epithelium, suggests that it may be more appropriate to target ookinetes located within midgut lumen as these are apparently more vulnerable. The location of ookinete losses within the midgut of naturally-infected mosquitoes has not been reported and the efficiency of the ookinete-to-oocyst transitions of laboratory strains of *P. falciparum* may be significantly different from field isolates (Vaughan, 2007). However, the overall ookinete-to-oocyst transition of the *P. falciparum* clone 3D7A in *A. stephensi* is only marginally less efficient (11% parasite survival) than that reported for field isolates of *P. falciparum* in laboratory-reared *A. gambiae* (16–84% of the ookinetes forming within the bloodmeal successfully transform into oocysts) (Gouagna et al., 1998, 2004a,b; Okech et al., 2004a,b).

Gupta et al. (2009) recently reported the characterisation in *A. gambiae* of a “late phase” anti-plasmodial immune response acting during oocyst development, concluding that oocysts are neither “hidden” nor unaffected by the mosquito’s immune system. In contrast, there was no evidence of *P. falciparum* oocyst mortality in *A. stephensi*, indicating that anti-plasmodial immune responses, if active, have no effect on parasite survival in our experimental system. Other studies using field isolates of *P. falciparum* have observed only low levels of oocyst mortality (2.7–16.7%) in *A. gambiae* (Gouagna et al., 1998, 1999, 2004a), while very high levels of oocyst mortality have been reported in field-collected *Anopheles arabiensis* (81.5%) (Awono-Ambene and Robert, 1998). Together with other studies describing inhibited oocyst development (Hunninen, 1953; Weathersby and McCroddan, 1982; Gonzalez-Ceron et al., 2001; Grieco et al., 2005), the importance (if any) of mosquito immune responses against oocysts is likely to vary considerably between different malaria parasite-mosquito vector combinations.

In summary, we have identified the endoperitrophic space as the site of *A. albimanus* refractoriness to infection with *P. falciparum* clone 3D7A, as well as the location of the majority of ookinete losses in the highly susceptible *A. stephensi*. Ookinete loss within the endoperitrophic space may be a general phenomenon, which also accounts for the refractoriness of *A. albimanus* to other non-human primate and rodent malaria parasite species (Omar, 1968b; Bafort, 1971; Nayar and Young, 1984; Vaughan et al., 1991, 1994a; Frischknecht et al., 2006). These findings highlight the importance of events within the endoperitrophic space as determinants of ookinete infection of the mosquito. Recent studies of malaria-mosquito interactions have understandably focused on ookinete invasion of the midgut epithelium while the endoperitrophic space, perhaps due to the considerable experimental challenges confronting in situ investigation of this midgut compartment, has been neglected and its potential importance overlooked. Research priorities for future studies should be to determine the mechanism(s) of ookinete loss within the endoperitrophic space in both susceptible and refractory mosquito species, and to estimate the contribution of different midgut compartments to ookinete losses that occur in the field.

## Figures and Tables

**Table 1:** Summary of the level of *Plasmodium falciparum* clone 3D7A oocyst infection in *Anopheles albimanus* and *Anopheles stephensi* at day 10 post-bloodfeeding (pbf), observed in 22 independent paired experimental feeds.

	*A. albimanus*	*A. stephensi*
Total number of mosquitoes examined	553	645
Total number of infected mosquitoes	0	512
Total number of oocysts observed	0	14,404
Mean number of mosquitoes examined per experimental feed (range)	25.1 (16–62)	29.3 (16–64)
Median % prevalence of oocyst infection per experimental feed (range)	0.0 (0.0)	84.4 (37.5–100.0)
Average median intensity of oocyst infection per infected mosquito per experimental feed (range)	–	18.5 (3.0–80.0)

**Table 2:** Comparison of the prevalence and intensity of early and late *Plasmodium falciparum* clone 3D7A oocyst infection in *Anopheles albimanus* and *Anopheles stephensi*.

		*A. albimanus*			*A. stephensi*			*P* value^e^
Experiment	Time (pbf)^a^	*n*^b^	Prevalence^c^	Intensity^d^	*n*^b^	Prevalence^c^	Intensity^d^	
1	2	20	0.0	0.0	20	95.0	21.5	<0.0001
	10	24	0.0	0.0	24	95.8	23.5	<0.0001
2	2	27	0.0	0.0	20	95.0	38.5	<0.0001
	10	32	0.0	0.0	34	94.1	45.5	<0.0001
3	2	24	0.0	0.0	22	81.8	11.5	<0.0001
	10	40	0.0	0.0	40	90.0	16.5	<0.0001

a Time post-bloodfeeding (pbf) in days.

b The number of midguts examined.

c The percentage of midguts examined containing at least one oocyst stage malaria parasite.

d The median number of oocysts per midgut.

e The prevalence and intensity of oocyst infection were compared between the two mosquito species, respectively, using a two-tailed Fisher’s exact test and two-tailed Mann–Whitney *U* test (*z*_0.05, 1.960_ < −5.397 in all instances). The *P* values indicated apply to the results of both tests.

**Table 3:** Comparison of the numbers of different stages of the *Plasmodium falciparum* clone 3D7A observed over time (h post-bloodfeeding, h pbf) in different locations within the midguts of *Anopheles albimanus* and *Anopheles stephensi* in histological sections. Data shown are pooled from two independent experimental feeds.

	*A. albimanus*					*A. stephensi*				
Time (h pbf)	*n*^a^	*n* Sections examined	OOK PM/EPS/MV^b^	OOK MGE^c^	OOC^d^	*n*^a^	*n* Sections examined	OOK PM/EPS/MV^b^	OOK MGE^c^	OOC^d^
24	2	853	1^e^	0	0	3	1,263	38	19	2
28	3	968	1^e^	0	0	4	1,579	129	48	31
32	7	1,591	1^e^	0	0	5	1,356	36	48	60
36	2	601	0	0	0	2	672	4	3	30
40	1	302	0	0	0	2	577	18	9	53
44	2	460	0	0	0	2	516	0	1	16
Overall	17	4,775	3	0	0	18	5,963	225	128	192

a The number of midguts examined.

b OOK PM/EPS/MV = extracellular ookinetes located either within the peritrophic matrix (PM), the ectoperitrophic space (EPS) or in the microvillar brush border (MV) of the midgut epithelium (MGE).

c OOK MGE = intra- or intercellular ookinetes located within the MGE.

d OOC = extracellular oocysts on the basal surface of the MGE.

e These three ookinetes were located on the internal bloodmeal (endoperitrophic) side of the PM.
